# Hypoxia Reduces Tau Aggregation Caused by Mitochondrial Bio-energetic Deficits

**DOI:** 10.1093/geroni/igaf122.3605

**Published:** 2025-12-31

**Authors:** Sushila Thapa, Raghbendra Dutta, Esther Ojumah, Isaiah Smith, Matt Kaeberlein, Anthony Grillo

**Affiliations:** University of Cincinnati, Cincinnati, Ohio, United States; University of Cincinnati, University of Cincinnati, Ohio, United States; University of Cincinnati, Cincinnati, Ohio, United States; University of Cincinnati, CIncinnati, Ohio, United States; Optispan, Optispan, Washington, United States; University of Cincinnati, University of Cincinnati, Ohio, United States

## Abstract

Tauopathies are marked by abnormal phosphorylation of the tau protein and are often linked to mitochondrial dysfunction. Among mitochondrial complexes, Complex I impairment has been associated with enhanced tau pathology, but the exact underlying mechanism remains unknown. Our study examines whether hypoxia can modulate tau phosphorylation and neuroinflammatory responses in models of mitochondrial and tau pathology. In Ndufs4 knockout (KO) mice, a model of primary Complex I deficiency, abnormally high brain oxygen tensions contribute to disease progression. Chronic exposure to a reduced oxygen environment (hypoxia) normalized these elevated oxygen levels, significantly decreasing astrogliosis and microglial activation. Additionally, hypoxia-treated Ndufs4 KO mice exhibited a reduction in hyperphosphorylated tau, suggesting that oxygen normalization mitigates tau pathology in the context of mitochondrial dysfunction. In contrast, tauopathy P301S mutant mice showed reduced astrogliosis and microglial activation under hypoxia, but tau phosphorylation remained unaltered. We further investigated tau expression in human neuroblastoma cell treated with a Complex I inhibitor, and found increased tau phosphorylation, reinforcing the link between mitochondrial impairment and tau pathology. Although the precise mechanisms remain to be clarified, our findings suggest that hypoxia may activate adaptive cellular pathways that mitigate mitochondrial dysfunction and influence tau phosphorylation. These results highlight hypoxia as a potential therapeutic strategy for tauopathies associated with mitochondrial deficits.

